# Supplementary material for: Saliva urea nitrogen for detection of kidney disease in adults: A meta-analysis of diagnostic test accuracy
Source: PLoS One. 2025 May 29;20(5):e0324251. doi: 10.1371/journal.pone.0324251 (PMC12121763; doi:10.1371/journal.pone.0324251)
Supplement: S2 Table — (DOCX) [file pone.0324251.s002.docx]

**S2 Table. Detailed search strategy for each database**

| **Database** | **Search strategy** |
| --- | --- |
| PubMed  <https://pubmed.ncbi.nlm.nih.gov> | ("renal disease" OR "renal injury" OR "kidney injury" OR "renal diseases" OR "renal failure" OR "renal function" OR "kidney diseases" OR "kidney disease" OR "renal insufficiency" OR "kidney insufficiency" OR "renal impairment") AND (" saliva" OR "salivary biomarkers" OR "salivary creatinine" OR "saliva urea" OR "saliva urea nitrogen"))  ("renal disease"[All Fields] OR "renal injury"[All Fields] OR "kidney injury"[All Fields] OR "renal diseases"[All Fields] OR "renal failure"[All Fields] OR "renal function"[All Fields] OR "kidney diseases"[All Fields] OR "kidney disease"[All Fields] OR "renal insufficiency"[All Fields] OR "kidney insufficiency"[All Fields] OR "renal impairment"[All Fields]) AND ("saliva"[All Fields] OR "salivary biomarkers"[All Fields] OR "salivary creatinine"[All Fields] OR "saliva urea"[All Fields] OR "saliva urea nitrogen"[All Fields]) |
| PubMed Central  <https://www.ncbi.nlm.nih.gov/pmc/> | (“Kidney Diseases” OR “Kidney Disease” OR “Chronic Kidney Disease”  OR “Chronic Renal Disease” OR “Renal Insufficiencies” OR  “Kidney Insufficiency” OR “Renal Function” OR “Kidney Function”  OR “Renal Failure” OR “Renal Injury”) AND ("Salivary Urea Nitrogen" OR “Salivary Creatinine”  OR “Salivary Urea” OR “Salivary Biomarkers”) |
| Scopus  <https://www.scopus.com/home.uri> | (“Kidney Diseases” OR “Kidney Disease” OR “Chronic Kidney Disease”  OR “Chronic Renal Disease” OR “Renal Insufficiencies” OR  “Kidney Insufficiency” OR “Renal Function” OR “Kidney Function”  OR “Renal Failure” OR “Renal Injury”) AND ("Salivary Urea Nitrogen" OR “Salivary” OR “Salivary Creatinine”  OR “Salivary Urea” OR “Salivary Biomarkers”) |
| Science Direct  <https://www.sciencedirect.com> | ("renal diseases" OR "kidney injury" OR "renal failure" OR "kidney diseases" OR "renal insufficiency") AND ("saliva” OR salivary biomarkers" OR "salivary creatinine “OR "saliva urea ") |
| Web of Science  <http://apps.webofknowledge.com/> | (“Kidney Diseases” OR “Kidney Disease” OR “Chronic Kidney Disease”  OR “Chronic Renal Disease” OR “Renal Insufficiencies” OR  “Kidney Insufficiency” OR “Renal Function” OR “Kidney Function”  OR “Renal Failure” OR “Renal Injury”) AND ("Salivary Urea Nitrogen" OR “Salivary Creatinine”  OR “Salivary Urea” OR “Salivary Biomarkers”) |
