# Supplementary material for: Saliva urea nitrogen for detection of kidney disease in adults: A meta-analysis of diagnostic test accuracy
Source: PLoS One. 2025 May 29;20(5):e0324251. doi: 10.1371/journal.pone.0324251 (PMC12121763; doi:10.1371/journal.pone.0324251)
Supplement: S3 Table — (DOCX) [file pone.0324251.s005.docx]

**S3 Table. Risk of bias and quality/certainly assessments for included studies.**

| Critical appraisal questions | Raimann et al. 2011 | Silva et al. 2014 | Raimann et al. 2016 | Evans et al. 2017 | Silva et al. 2018 | Evans et al. 2018 | Evans et al. 2020 |
| --- | --- | --- | --- | --- | --- | --- | --- |
| Domain 1: Patient Selection | | | | | | | |
| Was a consecutive or random sample of patients enrolled? | No | Unclear | Unclear | Yes | Unclear | Yes | Yes |
| Was a case control design avoided? | Yes | Yes | Yes | Yes | Yes | Yes | Yes |
| Did the study avoid inappropriate exclusions? | Yes | Yes | No | Yes | Yes | Yes | Yes |
| Domain 2: Index test | | | | | | | |
| Were the index test results interpreted without the knowledge of the reference standard? | Yes | Yes | No | Unclear | Unclear | Unclear | Yes |
| If a threshold was used, was it pre-specified? | Yes | Yes | Yes | Yes | Yes | Yes | Yes |
| Domain 3: Reference Standard | | | | | | | |
| Is the reference standard likely to correctly classify the target condition? | Yes | Yes | Yes | Yes | Yes | Yes | Yes |
| Were the reference standard results interpreted without knowledge of the results of the index test? | Yes | Yes | No | Yes | Unclear | Yes | Yes |
| Domain 4: Flow and Timing | | | | | | | |
| Was there an appropriate interval between index tests and reference standard? | Yes | Unclear | Yes | Yes | Yes | Yes | Yes |
| Did all patients receive a reference standard? | Yes | Unclear | Yes | Yes | Yes | Yes | Yes |
| Did all patients receive the same reference standard? | Yes | Yes | Yes | Yes | Yes | Yes | Yes |
| Were all patients included in the analysis? | Yes | Yes | No | Yes | Yes | Yes | Yes |
