# Supplementary material for: Saliva urea nitrogen for detection of kidney disease in adults: A meta-analysis of diagnostic test accuracy
Source: PLoS One. 2025 May 29;20(5):e0324251. doi: 10.1371/journal.pone.0324251 (PMC12121763; doi:10.1371/journal.pone.0324251)
Supplement: S4 Table — PPV: positive predictive value; NPV: negative predictive value; LR + : positive likelihood ratio; LR-: negative likelihood ratio; DOR: diagnostic odds ratio; CI: Confidence Interval. (DOCX) [file pone.0324251.s006.docx]

**S4 Table. Diagnostic test performance of individual studies that used salivary urea nitrogen.**

| Author, Year | No. | Sensitivity (95% CI) | Specificity  (95% CI) | PPV  (95% CI) | NPV  (95% CI) | LR+  (95% CI) | LR-  (95% CI) | DOR  (95% CI) |
| --- | --- | --- | --- | --- | --- | --- | --- | --- |
| Raimann, 2016 | 1 | 0.96 (0.82 to 0.99) | 0.90 (0.60 to 0.98) | 0.96 | 0.90 | 9.63 (1.50 to 61.91) | 0.04 (0.01 to 0.28) | 234.00 (13.22 to 4141.89) |
| Silva, 2014 | 2 | 0.91 (0.62 to 0.98) | 0.79 (0.62 to 0.89) | 0.59 | 0.96 | 4.29 (2.16 to 8.49) | 0.12 (0.02 to 0.75) | 37.14 (4.04 to 341.50) |
| Silva, 2018 | 3 | 0.67 (0.48 to 0.81) | 0.98 (0.91 to 1.00) | 0.95 | 0.87 | 39.33 (5.53 to 279.66) | 0.34 (0.20 to 0.58) | 116.00 (13.75 to 978.65) |
| Raimann, 2011 | 4 | 0.86 (0.78 to 0.98) | 0.89 (0.72 to 0.96) | 0.96 | 0.65 | 7.74 (2.66 to 22.57) | 0.16 (0.09 to 0.26) | 49.23 (12.95 to 187.21) |
| Evans, 2018 | 5 | 0.13 (0.06 to 0.27) | 0.97 (0.95 to 0.99) | 0.42 | 0.88 | 4.80 (1.60 to 14.38) | 0.90 (0.79 to 1.01) | 5.36 (1.61 to 17.82) |
| Evans, 2020 | 6 | 0.30 (0.42 to 0.53) | 0.91 (0.93 to 0.97) | 0.91 | 0.32 | 3.42 (1.74 to 6.71) | 0.78 (0.60 to 2.82) | 4.37 (2.56 to 7.47) |
| Evans, 2017 | 7 | 0.72 (0.64 to 0.79) | 0.87 (0.84 to 0.90) | 0.58 | 0.93 | 5.57 (4.42 to 7.02) | 0.32 (0.25 to 0.42) | 17.26 (11.19 to 26.62) |

PPV: positive predictive value; NPV: negative predictive value; LR+: positive likelihood ratio; LR-: negative likelihood ratio; DOR: diagnostic odds ratio; CI: Confidence Interval
