# Supplementary material for: Saliva urea nitrogen for detection of kidney disease in adults: A meta-analysis of diagnostic test accuracy
Source: PLoS One. 2025 May 29;20(5):e0324251. doi: 10.1371/journal.pone.0324251 (PMC12121763; doi:10.1371/journal.pone.0324251)
Supplement: S5 Table — (DOCX) [file pone.0324251.s007.docx]

## **S5 Table. Diagnostic accuracy measures for sCr and BUN subgroup analyses (with 95% confidence intervals)**

## **A. sCr subgroup analysis**

| Metric | Estimate (95% CI) | Interpretation |
| --- | --- | --- |
| Sensitivity | 0.44 (0.38–0.49) | Proportion of true positives correctly identified |
| Specificity | 0.96 (0.95–0.98) | Proportion of true negatives correctly identified |
| Positive Likelihood Ratio (PLR) | 11.00 (7.90–15.32) | A positive result is ~11× more likely in those with kidney disease |
| Negative Likelihood Ratio (NLR) | 0.58 (0.52–0.65) | A negative result occurs in ~58% of cases with the disease |
| Diagnostic Odds Ratio (DOR) | 18.86 (15.19–23.57) | Overall diagnostic ability of SUN vs. sCr |

## **B. BUN subgroup analysis**

| Metric | Estimate (95% CI) | Interpretation |
| --- | --- | --- |
| Sensitivity | 0.83 (0.69–0.91) | Proportion of true positives correctly identified |
| Specificity | 0.88 (0.78–0.94) | Proportion of true negatives correctly identified |
| Positive Likelihood Ratio (PLR) | 7.1 (3.7–13.8) | A positive result is ~7× more likely in those with kidney disease |
| Negative Likelihood Ratio (NLR) | 0.19 (0.10–0.36) | A negative result occurs in ~19% of cases with the disease |
| Diagnostic Odds Ratio (DOR) | 37 (15–91) | Overall diagnostic ability of SUN vs. BUN |
